# Comparison of a high-carbohydrate and high-protein breakfast effect on plasma ghrelin, obestatin, NPY and PYY levels in women with anorexia and bulimia nervosa

**DOI:** 10.1186/1743-7075-9-52

**Published:** 2012-06-08

**Authors:** Dana Sedlackova, Jana Kopeckova, Hana Papezova, Vojtech Hainer, Hana Kvasnickova, Martin Hill, Jara Nedvidkova

**Affiliations:** 1 Institute of Endocrinology, Narodni 8, 116 94, Prague 1, Czech Republic; 2 First Faculty of Medicine, Charles University, Prague, Czech Republic; 3 Department of Anthropology and Human Genetics, Charles University, Prague, Czech Republic; 4 Faculty of Science, Department of Anthropology and Human Genetics, Charles University, Vinicna 7, 128 44, Prague 2, Czech Republic; 5 First Faculty of Medicine, Psychiatric Department, Ke Karlovu 11, 120 00, Prague 2, Czech Republic

**Keywords:** Ghrelin, Obestatin, NPY, PYY, Anorexia nervosa, Bulimia nervosa, High-carbohydrate breakfast, High-protein breakfast

## Abstract

**Background:**

The present study investigated plasma levels of gut-brain axis peptides ghrelin, obestatin, NPY and PYY after consumption of a high-carbohydrate (HC) and high-protein (HP) breakfast in patients with anorexia nervosa, bulimia nervosa and in healthy controls. These peptides play an important role in regulation of energy homeostasis and their secretion is disturbed under condition of eating disorders. As various types of consumed macronutrients may induce different plasma hormone responses, so we examined these responses in women patients with eating disorders and compared them with those of healthy controls.

**Methods:**

We examined plasma hormone responses to HC and HP breakfast in patients with AN (n = 14; age: 24.6 ± 1.8 years, BMI: 15.3 ± 0.7), BN (n = 15; age: 23.2 ± 1.7 years, BMI: 20.5 ± 0.9) and healthy controls (n = 14; age: 24.9 ± 1.4 years, BMI: 21.1 ± 0.8). Blood samples were drawn from the cubital vein, the first blood drawn was collected before meal, and then 30, 60, 90, 120 and 150 min after breakfast consumption. Plasma hormone levels were determined by commercially available RIA kits.

**Results:**

Fasting and postprandial plasma obestatin levels were significantly increased in both AN and BN patients, while plasma ghrelin levels were significantly increased in AN patients only. After breakfast consumption, plasma levels of ghrelin and obestatin decreased, although they were still above the range of values of healthy controls. Fasting NPY plasma levels were significantly increased in AN and BN patients and did not change postprandially. Fasting PYY levels were comparable in AN, BN and healthy controls, but postprandially significantly increased after HP breakfast in AN and BN patients. Different reactions to breakfast consumption was found for ghrelin and PYY among investigated groups, while for obestatin and NPY these reactions were similar in all groups.

**Conclusions:**

Significant increase of obestatin and NPY in AN and BN patients may indicate their important role as the markers of eating disorders. Different reactions of ghrelin and PYY to breakfast consumption among groups suggest that role of these hormones in regulation of energy homeostasis can be adjusted in dependence to acute status of eating disorder.

## Background

Food intake and appetite are controlled by hypothalamus, where nucleus arcuatus (ARC) through interaction of peripheral hormonal and metabolic signals control all processes relating to energy balance. Peptides of gut-brain axis play a pivotal role in regulation of energy homeostasis and their secretion is disturbed under condition of eating disorders, including anorexia nervosa (AN) and bulimia nervosa (BN). These psychiatric and somatic diseases are characterized by abnormal eating behaviour and imbalance in energy homeostasis. Neuropeptide Y (NPY) has a number of important functions in regulation of appetite and energy homeostasis [1]. NPY neurons in the hypothalamic ARC play a central role in stimulation of feeding, they sense and integrate peripheral and central signals, including ghrelin and leptin [2]. Ghrelin is a peptide produced mainly by stomach, which as appetite-stimulating hormone transmits changes in food intake to the central nervous system [3]. The effect of ghrelin is related to the antagonism of the inhibitory effect of leptin on hypothalamic NPY production [4-6]. A novel peptide hormone obestatin, derived from the same gene as ghrelin, has been initially postulated to antagonize ghrelin actions on energy homeostasis and gastrointestinal functions [7-9], however the most of subsequent studies could not confirm its reported anorexigenic effects [10-13]. Plasma levels of these hormones, both fasting and postprandial, were investigated in AN and BN patients. Most of previous studies reported increased fasting plasma ghrelin levels in AN patients [3,14-17] and recent studies also presented increased fasting plasma obestatin levels in these patients [17,18]. In BN patients were reported either no changes in fasting ghrelin or increased plasma ghrelin levels, compared to healthy controls [15,19,20]. Monteleone et al. (2008) did not find any increase in fasting obestatin plasma levels in BN patients [17]. However, in our previous study, we found increased fasting plasma ghrelin levels only in AN patients while obestatin levels were elevated in both group of patients with AN and BN [21]. Peptide YY (PYY) is a member of the pancreatic polypeptide family, which has been reported to reduce food intake [22]. It is suggested that peripheral PYY acts as a satiety signal regulating the termination of individual meals, partially by decreasing the production of the hunger-stimulating peptide ghrelin [17]. Fasting PYY levels have been reported to be low in healthy people [23,24], normal [25] or increased [26-28] in AN patients and normal in BN patients [20,23]. Leptin is a peptide hormone, secreted mainly by adipose tissue, which influences long-term energy homeostasis. Decreased leptin levels were measured in AN patients with undernutrition [19,29].

All introduced hormones are involved in regulation of food intake and energy homeostasis, either in acute (ghrelin, obestatin, PYY) or long-term changes (leptin, NPY). The effect of specific macronutrients (carbohydrates, proteins, fat) on secretion of above mentioned hormones has been partially investigated in humans. However, results reported in individual studies are very different, especially in studies aimed to patients with eating disorders. Prince et al. compared the results of studies concerned to gut hormone levels in patients with eating disorders, with general conclusion that these patients had higher baseline concentrations of ghrelin and PYY. No differences were found in release of these hormones to a standardized test meal, when compared to healthy controls [30].

To our knowledge, no study has measured simultaneously pre- and postprandial secretion of ghrelin, obestatin, NPY, PYY and leptin in patients with AN and BN. Therefore the aim of present study was to investigate plasma levels of these hormones after consumption of two meals with different content of macronutrients (high-protein and high-carbohydrate breakfast) in patients with AN, BN and healthy women. We investigated if plasma hormone responses are different after consumption of high-protein and high-carbohydrate breakfast within the observed groups. Simultaneously, we compared if plasma hormone responses to the same HC/HP breakfast differs among patients with AN, BN and healthy controls.

## Methods

### Subjects

This study was performed in accordance with the Declaration of Helsinki and was approved by Ethic Committee of Institute of Endocrinology in Prague. All participants undersigned informed consent prior to the study. Fourteen patients with AN, both restrictive and purgative type (age: 24.6 ± 1.8 years, BMI: 15.3 ± 0.7), fifteen women with BN (age: 23.2 ± 1.7 years, BMI: 20.5 ± 0.9) and fourteen healthy controls (age: 24.9 ± 1.4 years, BMI: 21.1 ± 0.8 kg/m^2^) were enrolled in the study. Healthy controls (C) were recruited from university students, age-matched to AN and BN patients. They had no history of eating disorders, had normal electrocardiogram (ECG), blood count, liver and renal functions. Blood tests and physical examination were conducted before the test. All healthy women had regular menstrual cycle and were in follicular phase of the cycle at the time of study. AN and BN patients were diagnosed according to the 4^th^ edition of the Diagnostic and Statistical Manual of Mental Disorders, American Psychiatric Association, 1994. All AN and BN patients were clinically stable and in relatively good health, except for their eating disorder and amenorrhea. All patients were investigated after 1 week of hospitalization at the Department of Psychiatry at Charles University, Prague. Blood tests conducted before initiation of the study confirmed normal values for blood count, fasting blood glucose, liver and renal functions.

Participants were recommended to avoid vigorous physical activity during the 14-hour period before blood samplings. All subjects consumed a standardized dinner at 6.00 PM and were then asked to fast overnight. Reported duration of sleep in the night preceding blood sampling was comparable in all studied groups. All participants were admitted to the Institute of Endocrinology at 7.30 AM. The study lasted about 3.5 hours and the protocol consisted of high-carbohydrate breakfast consumption and blood withdrawals. One week later, all patients were investigated again and they received high-protein breakfast with the same study protocol. Body composition was measured using method of bioimpedance [Tanita, Japan] together with other physical examination before the beginning of both breakfasts.

### Study design

Each participant received a high-carbohydrate breakfast (HC) with a total energy content of 1604 kJ, consisting of 81.9 g carbohydrates, 8.8 g proteins and 3.4 g fats in the form of a white bread roll (90 g) and strawberry jam (50 g) [21]. One week later, each participant received a high-protein breakfast (HP) with a total energy content of 1350 kJ, consisting of 41.3 g proteins, 16.3 g carbohydrates and 8.1 g fat in the form of a cottage cheese (150 g), chicken ham (75 g) and wheat store-bread (15 g). In addition, the subjects consumed 250 ml of fruit tea without sugar or other sweetener together with the meal. Participants had 15 minutes time- limit to consume their meal. Blood samples were drawn from the cubital vein using an intravenous cannula, the first blood drawn was collected before meal, and then 30, 60, 90, 120 and 150 min after breakfast consumption. Blood samples were collected into chilled polypropylene tubes containing Na_2_EDTA and antilysin. Plasma was immediately separated by 15-min centrifugation at 5°C and stored at −70°C until being assayed [21].

### Analytical measurements

Total plasma ghrelin and NPY were determined by commercially available RIA kits (Linco Research, Inc., St. Charles, Missouri, U.S.A.). The intra- and interassay for total ghrelin was 6.4% and 16.3%, sensitivity was 93 pg/mL, for NPY the intra- and interassay was 5.0% and 8.4% respectively, sensitivity was 3 pmol/L. Plasma obestatin was measured by a commercial RIA kit (Phoenix Pharmaceuticals Inc., Belmont, CA, U.S.A.), the intra- and interassay variability was 5.0% and 14.2%, respectively, sensitivity was 50 pg/mL. Plasma PYY was determined by commercially available RIA kit (Linco Research, Inc., St. Charles, Missouri, U.S.A.). The intra- and interassay for total PYY was 5.8% and 13.9%, sensitivity was 72 pg/mL.

### Statistical data analysis

The dependence of metric variables on factors was evaluated using repeated measures ANOVA model consisting of subject factor, repeated and non-repeated factors and interaction between factors. Least significant difference multiple comparisons followed the ANOVA testing. The level of statistical significance P < 0.05 was chosen for both ANOVA and multiple comparisons. Due to non-Gaussian data distribution in all dependent variables these underwent power transformations to attain distributional symmetry and a constant variance both in the data and residuals. Data transformation and analysis of variance was performed using Statgraphics Centurion version XV software (Statpoint Inc, Herndon, Virginia, USA). The relationships between variables were evaluated using multivariate regression after transformation of variables to symmetry and constant variance. The statistical software SIMCA v. 12.0 from Umetrics (Umeå, Sweden) was used for data analysis.

## Results

**Figure 1:**
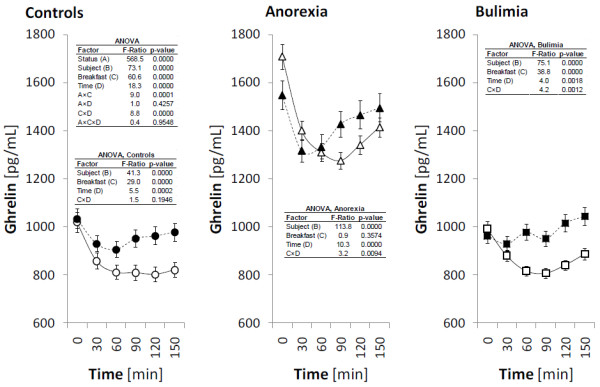
**Plasma levels of ghrelin during the meal test.** The circles, triangles and squares with error bars represent the retransformed means with their 95% confidence intervals for controls (C), patients suffering with anorexia nervosa (AN), and patients suffering with bulimia nervosa (BN), respectively as evaluated by repeated measures ANOVA followed by least significant difference multiple comparisons. For the details see Statistical data analysis. High-carbohydrate breakfast is marked by empty symbol (○, ▵, □), high-protein breakfast is marked by full symbol (●, ▲, ■).

**Figure 2:**
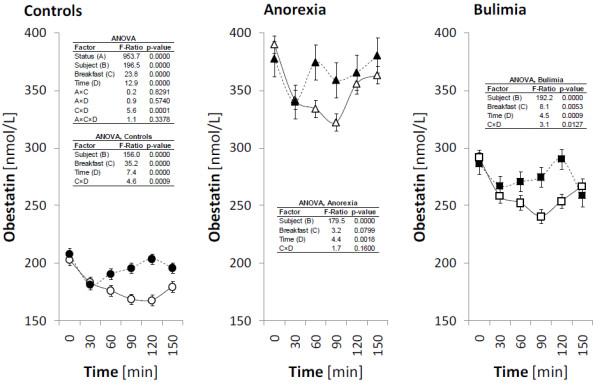
**Plasma levels of obestatin during the meal test.** The circles, triangles and squares with error bars represent the retransformed means with their 95% confidence intervals for controls (C), patients suffering with anorexia nervosa (AN), and patients suffering with bulimia nervosa (BN), respectively as evaluated by repeated measures ANOVA followed by least significant difference multiple comparisons. For the details see Statistical data analysis. High-carbohydrate breakfast is marked by empty symbol (○, ▵, □), high-protein breakfast is marked by full symbol (●, ▲, ■).

**Figure 3:**
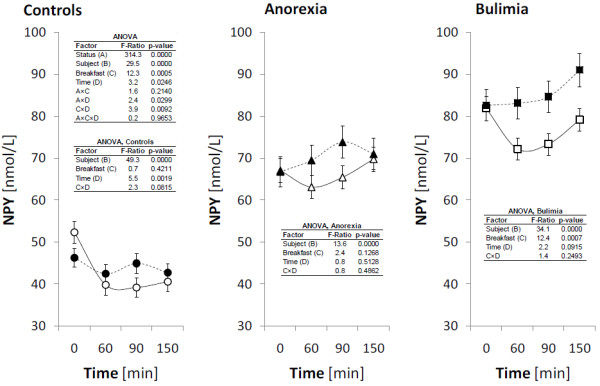
**Plasma levels of NPY during the meal test.** The circles, triangles and squares with error bars represent the retransformed means with their 95% confidence intervals for controls (C), patients suffering with anorexia nervosa (AN), and patients suffering with bulimia nervosa (BN), respectively as evaluated by repeated measures ANOVA followed by least significant difference multiple comparisons. For the details see Statistical data analysis. High-carbohydrate breakfast is marked by empty symbol (○, ▵, □), high-protein breakfast is marked by full symbol (●, ▲, ■).

**Figure 4:**
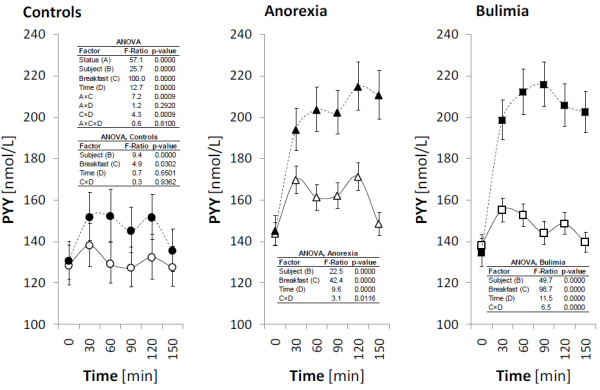
**Plasma levels of PYY during the meal test.** The circles, triangles and squares with error bars represent the retransformed means with their 95% confidence intervals for controls (C), patients suffering with anorexia nervosa (AN), and patients suffering with bulimia nervosa (BN), respectively as evaluated by repeated measures ANOVA followed by least significant difference multiple comparisons. For the details see Statistical data analysis. High-carbohydrate breakfast is marked by empty symbol (○, ▵, □), high-protein breakfast is marked by full symbol (●, ▲, ■).

The fasting plasma levels of ghrelin, obestatin, NPY and PYY and their postprandial responses after high-carbohydrate (HC) and high-protein (HP) breakfast consumption are presented in Figures 1, 2, 3 and 4 for all investigated groups.

### Ghrelin

Fasting and postprandial plasma ghrelin levels were significantly increased in AN patients compared to the controls and BN patients. After both types of breakfast, plasma ghrelin levels significantly decreased, with minimum at 90 min after HC breakfast and 30 min after HP breakfast. Factor “breakfast” was not significant in AN patients, but interaction “breakfast” x “time (stage of the meal test)” showed different course of ghrelin in time after HC and HP breakfast. In BN patients, fasting and postprandial levels of ghrelin were comparable to values of healthy controls. After both types of breakfast, plasma ghrelin levels decreased, however this decrease was significant only after HC breakfast. Minimum ghrelin values were reached 90 min after HC breakfast and 30 min after HP breakfast (equally, as in AN patients). The course of ghrelin after both types of breakfast was different after HC and HP breakfast in BN patients. In healthy control women, plasma ghrelin levels significantly decreased after both types of breakfast with minimum at 120 min after HC breakfast and 60 min after HP breakfast. The factor “breakfast” was significant. As documented by significant interaction “status” x “breakfast” in overall ANOVA, the ghrelin reaction was different after HC and HP breakfast consumption among investigated groups.

### Obestatin

Fasting plasma obestatin levels were found significantly increased in both AN and BN patients compared to the controls. The highest basal values were observed in AN patients, similarly as plasma ghrelin values. In these patients plasma obestatin levels decreased significantly after both types of breakfast, with minimum at 90 min after HC and 30 min after HP breakfast. Neither factor “breakfast” nor interaction “breakfast” x “time” were significant in AN patients, and therefore no difference was found in ghrelin reaction in time after HC and HP breakfast consumption. In BN patients, plasma obestatin levels decreased significantly after both types of breakfast, with minimum values at 90 min after HC breakfast and 150 min after HP breakfast. As documented by significant factor “breakfast“ and interaction “breakfast” x “time”, course of obestatin after both types of breakfast was different in BN patients. In healthy control group, plasma obestatin levels also significantly decreased after both HC and HP breakfast consumption and course of obestatin was different in time. The obestatin reaction after HC and HP breakfast was similar in all groups, although plasma levels had different range of values, as documented by interaction “status” x “breakfast” in overall ANOVA.

### NPY

Fasting and postprandial plasma levels of NPY were significantly increased in AN and BN patients compared to the controls. No changes were found postprandially in AN patients after both types of breakfast, but in BN patients and healthy controls plasma NPY decreased after HC breakfast. After HP breakfast consumption no significant changes were found. However, as indicated by interaction “breakfast” x “time”, the course of NPY after HC and HP breakfast consumption was not significantly different in all investigated groups. Also no differences were found in NPY reaction to breakfasts consumption among investigated groups.

### PYY

Fasting plasma levels of PYY were found in similar range of values for all investigated groups. However, significant differences were found in postprandial responses of PYY to HC and HP breakfast in patients with AN and BN compared to the controls. In AN and BN groups, plasma PYY levels reached significantly higher values after HP breakfast compared to HC breakfast, with maximum in 120 min for AN and 90 min for BN. In healthy control group, postprandial increase was also observed after HP breakfast, however this increase was not significant and reached values were significantly lower than plasma PYY levels in AN and BN groups. As documented by significant interaction “status” x “breakfast” in overall ANOVA, the PYY course after HC and HP breakfast was different among investigated groups.

### Leptin

Basal plasma levels of leptin were found significantly lower in AN and BN patients compared to healthy women. We found no changes in leptin levels postprandially either after HC or HP breakfast consumption in all investigated groups.

### Correlations

Status of anorexia nervosa was found to be positively correlated with plasma levels of ghrelin, obestatin, NPY and PYY and negatively correlated with BMI, percent of body fat and weight. Status of bulimia nervosa was found to be positively correlated with plasma levels of NPY, obestatin and PYY, and negatively with leptin levels.

## Discussion

This study was designed to investigate the plasma responses of ghrelin, obestatin, NPY and PYY after high-carbohydrate and high-protein breakfast consumption in AN and BN patients and in healthy women. We found fasting plasma obestatin levels significantly increased in AN and BN patients, while ghrelin levels were increased only in AN patients. Postprandially, we observed significant decrease in plasma ghrelin levels after both types of breakfast in all investigated groups. Different plasma response of ghrelin to HC and HP breakfast was observed in all investigated groups. Regarding obestatin levels, they also decreased postprandially after both types of breakfast in all investigated groups. In BN patients and healthy controls the reaction of obestatin was different to HC and HP breakfast, however in AN patients no difference was found.

Our results for fasting ghrelin and obestatin levels in AN patients and healthy controls are consistent with present findings of other authors. Recent studies showed increased plasma obestatin and ghrelin levels in AN patients [18,26,31]. Monteleone et al. (2008) found increased fasting plasma obestatin levels in AN patients, however in BN patients obestatin levels were comparable to healthy controls [17]. In our previous study, we demonstrated for the first time that plasma obestatin levels significantly decrease after consumption of a high- carbohydrate breakfast in a similar way as ghrelin levels in healthy women [32]. Results of present study correspond with our previous findings and were confirmed also for AN and BN patients, independently on the type of consumed breakfast. This positive relationship of obestatin and ghrelin in postprandial period indicates that these two cleavage products of preproghrelin act in a similar way to increase food intake. Comparison of hormone responses to HC and HP breakfast confirmed mostly different reaction of these hormones in time to the type of macronutrient. During comparison of groups, similar reactions to food intake were confirmed for obestatin in AN patients, BN patients and healthy women, however the values of plasma obestatin were different. Contrary, ghrelin responses to food intake were different among groups.

NPY plays a central role in stimulation of feeding and regulation of energy homeostasis. We found fasting plasma levels of NPY significantly higher in AN and BN patients, when compared to the healthy controls. This finding is partially in accordance with studies of other authors, where NPY levels were reported to be both increased [33,34] and decreased [35] in AN patients. Plasma NPY levels in our study showed no difference in time to HC or HP breakfast in all groups. Overall analysis for all groups showed that reaction of plasma NPY to HC and HP breakfast consumption was not significantly different among groups. To date, no study has reported any response of NPY to carbohydrate and protein meal in healthy controls or AN and BN patients, therefore we have no possibility to compare our findings. Production of leptin correlates positively with adipose tissue mass and circulating leptin levels, and thus reflects energy stores of organism. In our study fasting plasma leptin levels were significantly lower in AN and BN patients and no changes in were found postprandially.

Circulating PYY levels are low in the fasting state and rapidly increase postprandially when two forms, PYY1-36 and PYY3-36 are released to circulation. In our study we observed significant increase in postprandial plasma levels in AN and BN patients after HP breakfast, much higher than those in healthy controls. This suggests strong influence of macronutrient type to postprandial secretion of PYY in patients with eating disorders. These results partially correspond with study of Nakahara et al. (2007), where PYY3-36 plasma levels were increased after standard meal consumption in both AN patients and healthy controls [26]. Our results are also partly in accordance with review of Prince at al. (2009), where patients with eating disorders had higher baseline concentrations of PYY and ghrelin [30]. In contrast, in study of Stock et al. (2005) PYY increased significantly in the controls, but not in AN patients [36]. In BN patients, initial studies reported normal plasma levels of PYY. Recently, two independent research groups have reported a blunted PYY3-36 response to food ingestion in symptomatic BN women together with a decreased response of ghrelin [20,23]. Both studies have shown a negative correlation between meal-induced PYY increase and ghrelin decrease, confirming a negative interaction of PYY3-36 with ghrelin [17].

Correlations between status of eating disorder and investigated hormones were evaluated. As status of anorexia nervosa was found to be positively correlated with plasma levels of ghrelin, obestatin, NPY and PYY, we suppose that all these hormones are included in pathology of eating disorder and their levels are changed probably as a consequence of eating disorder. Status of bulimia nervosa was found to be positively correlated with plasma levels of NPY, obestatin and PYY and negatively with leptin levels, which suggest a lot of common with status of anorexia nervosa, at least regarding behavior of plasma hormones and disturbed regulation of energy homeostasis.

## Conclusions

In conclusion, we demonstrated that significant differences exist in hormonal responses of ghrelin, obestatin and PYY to a high-carbohydrate and high-protein breakfast consumption within investigated groups. Increased fasting plasma levels of NPY and obestatin were confirmed in AN and BN groups, which suggest a role of these hormones as a markers for eating disorders. PYY levels reached much higher values after high-protein breakfast in AN and BN patients, indicating an important role of ingested macronutrient to plasma levels of this hormone. Different reactions of ghrelin and PYY to breakfast consumption were found among investigated groups.

## Competing interests

The authors declare that there is no conflict of interests.

## Authors’ contributions

DS carried out research, contributed on analytical measurements and wrote the article. JK participated on research and analytical measurements. HP selected and provided hospitalized pacients with AN and BN suitable for the study. VH contributed in writing of the article. HK examined all participants prior to the study. MH performed statistical data analysis. JN designed research, led the grant project, contributed in writing of the article. All authors read and approved the final manuscript.
